# Supplementary material for: Parcellation-Based Connectivity Model of the Judgement Core
Source: J Pers Med. 2023 Sep 16;13(9):1384. doi: 10.3390/jpm13091384 (PMC10532823; doi:10.3390/jpm13091384)
Supplement: Supplementary file 1 [file jpm-13-01384-s001.zip › Supplement File S1_Revised.pdf]

Supplement S1: Parcellation Based Connectivity Model of the Judgement Core

Supplement S1, Table S1. Description of selected modality-based sub-analyses

Domain	Paradigm	Task Description
Moral	Moral Dilemma Decision	Participants must choose between opposing moral principles. Most commonly achieved by creating scenarios with opposing utilitarian and deontological choice alternatives. For an example, consider the often- used 'trolley-bridge' problem.
	Moral Evaluation	Must evaluate the moral integrity of a provided stimulus. The range of good- evil is frequently quantified with a point rating system.
Social	Trait Judgement	Asked to determine a specific quality of another person based on a sample of their past behaviour. For example, a story about a former interaction, or a vocal excerpt.
	Face Monitoring	Asked to determine a specific quality of another person based on their facial appearance. Most often participants are asked to judge how trustworthy or approachable an unknown other looks.
Interpersonal	Prisoners Dilemma	Two parties who are separated and unable to communicate must choose between cooperating or defecting. Mutual cooperation leads to greatest overall yield for both parties. If one participant defects and the other cooperates, the defector receives a greater yield than mutual cooperation. Mutual defecting leads to the smallest gain for each player out of all possible scenarios. These paradigms may be 'single-shot' where the participants play with the participants only once. Or iterated where they play a number of times with the same partner.

	Trust Game	Participants may play as trustee or trustor. The trustor possesses an initial sum of money. They may donate a portion of that sum to the trustee. The received portion will multiply in value before the trustee is given the opportunity to give back a portion to the original trustor or keep the earnings for themself. Can be played in both single-shot and iterated variations.
	Ultimatum Game	Participants play as either a prosper or responder.<br>Proposer is endowed with a starting sum of money, the quantity of which is known to both parties. The proposer suggests a division of this sum between themself and the responder. If the responder rejects neither party receives anything. If the responder accepts the funds are distributed accordingly. Can be played in both single-shot and iterated variations.
Risk Judgement	Balloon-analogue Risk Task	Balloon progressively inflates. Greater inflation provides greater winnings, however also increases the chance of 'popping' which will return nothing.<br>Participants must choose when to stop inflating the virtual balloon.
	Probabilistic Forced Choice	Forced to make a decision based on partially known probabilities.
	Simple Gamble Task	Presented with odds for a win/loss condition and asked to accept or reject.
	Binary Gamble Choice	Presented with odds for a pairing of winn/loss conditions.<br>Participants must choose between them.
	Wheel of Fortune	Odds are graphically represented on a wheel. Participants must choose to accept the offered gamble or choose between alternative gambles.

Supplement S1, Table S2. Behavioral analysis across moral, social, risky and interpersonal judgment paradigms. Activation likelihood estimation (ALE) maps were created as described in the text for each judgement paradigm and were further analyzed according in BrainMap. ALE coordinate data, consisting of anatomical coordinates most representative of each judgement paradigm, were analyzed using the activation coordinate experiment-wise search (ACES) on BrainMap to find the top 10 most similar behavioral experiments to each of these judgement paradigm coordinates in the BrainMap database.

Moral

BrainMap ID	Exp. Size	Coordinates Matching	Coordinate Similarity
5040017 1	22	6	30%
Name: Brain Activation Positively Related to Perceived Pain Intensity			
7060142 5	40	6	30%
Name: Go > NoGo			
8020052 5	2	6	30%
Name: Voluntary Saliva Swallow + Voluntary Finger Opposition			
8020052 6	3	6	30%
Name: Voluntary Tongue Elevation + Voluntary Finger Opposition			
5070162 3	14	5.9	29%
Name: Kanji Mental Recall (iMR) vs. Rest			
14050049 3	9	5.9	29%
Name: SWITCH, Healthy Controls			
8020071 1	10	5.4	27%
Name: Group Analysis for Right Hand Movement			
4040029 1	15	5.4	27%
Name: Working Memory, Schizophrenic Patients			
13030024 3	17	5.4	27%
Name: Non-Meaningful > Metaphoric			
60100148 5	6	5.3	27%
Name: Bimanual Asymmetric Finger Press vs. Unimanual Right Finger Press			

Social

BrainMap ID	Exp. Size	Coordinates Matching	Coordinate Similarity
5070070 4	24	5.6	31%
Name: Dose-Dependent Deactivations			
6110180 4	31	4.9	27%
Name: Deqi + Pain, Activations			
10060127 2	20	4.8	27%
Name: Healthy Controls > Depressed Patients, Encoding			
14070154 2	22	4.2	23%
Name: 1-Back minus 0-Back, Traumatic Brain Injury Patients > Orthopedic Injury Patients, Cluster A			
14100213 6	7	4.1	23%
Name: Other > high-level baseline, control fathers			
7020033 2	25	3.8	21%
Name: Familiar Faces, Controls			
7080199 5	4	3.7	21%
Name: Main Effect of Probability (Activations)			
9030059 2	8	3.7	21%
Name: Emotional > Neutral Pictures, Activations			
30291 1	4	3.5	20%
Name: Small-Large Rotations			
14100213 9	9	3.5	20%
Name: Other low > high-level baseline, ASD			

Risk			
**BrainMap ID**	**Exp. Size**	**Coordinates Matching**	**Coordinate Similarity**
11080094 1	16	3.1	22%
Name: High-Risk Positive Feedback > Low-Risk Positive Feedback in Healthy Young Adults			
12020014 4	23	3.1	22%
Name: (Increase, Negative Pictures > Maintain, Negative Pictures), Whole Group			
14050128 2	23	2.6	19%
Name: Pattern Matching > Fixation, Autism Group (AUT)			
14090207 5	16	2.6	19%
Name: Memory Selection Unique			
10080156 4	8	2.5	18%
Name: Go > No Go, Healthy Controls			
30261 1	20	2.4	17%
Name: CO2FM vs. CO2MP, Activations			
7070166 2	31	2.3	17%
Name: Anomalous Metaphor Sentences vs. Literal Sentences			
8110257 1	17	2.3	16%
Name: Painful - Non-Painful Facial Expressions			
14070154 2	22	1.9	14%
Name: 1-Back minus 0-Back, Traumatic Brain Injury Patients > Orthopedic Injury Patients, Cluster A			
14050047 3	8	1.9	13%
Name: Counting Stroop (cStroop), Control			

Interpersonal			
**BrainMap ID**	**Exp. Size**	**Coordinates Matching**	**Coordinate Similarity**
12020014 4	23	9.7	34%
Name: (Increase, Negative Pictures > Maintain, Negative Pictures), Whole Group			
10080156 4	8	8.5	30%
Name: Go > No Go, Healthy Controls			
11080094 1	16	8.5	30%
Name: High-Risk Positive Feedback > Low-Risk Positive Feedback in Healthy Young Adults			
8040095 1	12	8	29%
Name: (IS>S) > (IP>P), Brain Activations During Transitive Inference Condition, Normals			
14050128 2	23	6.8	24%
Name: Pattern Matching > Fixation, Autism Group (AUT)			
8110257 1	17	6.4	23%
Name: Painful - Non-Painful Facial Expressions			
7110330 2	35	5.7	20%
Name: Painful > Neutral, Alexithymia Patients			
8060158 3	15	5.7	20%
Name: Interaction of Utilitarian and Performance Feedback			
11010021 10	54	5.7	20%
Name: Correlation with Reward Probability			
7120387 1	20	5.7	20%
Name: High > Low Provocation			
