# Supplementary material for: Parcellation-Based Connectivity Model of the Judgement Core
Source: J Pers Med. 2023 Sep 16;13(9):1384. doi: 10.3390/jpm13091384 (PMC10532823; doi:10.3390/jpm13091384)
Supplement: Supplementary file 1 [file jpm-13-01384-s001.zip › Supplement File S2.pdf]

Supplement S2: Parcellation Based Connectivity Model of the Judgement Core

Supplement S2. Description of included studies with data points

**Subgroup = Moral**					
	**Author**	**Participants**	**Paradigm**	**BOLD Contrast**	**MNI Coordinates**
1	Heinzelmann, 2020	27 (18F; 9M)	Evaluation of visually depicted moral scenario	Task>perceptual control (speed of action).	0 50 13<br>-45 23 -11<br>-30 14 -17<br>30 20 -17<br>-45 -61 28<br>-45 5 -29<br>-6 -52 22
2	Cunningham, 2004	20 (gender ratio undeclared)	Evaluation of moral goodness of word concepts (e.g. recycling)	Task>semantic categorisation task (is the shown word abstract or concrete in meaning)	0 28 -8<br>-8 52 20<br>12 56 8<br>16 -8 -20<br>-4 -56 28<br>64 -12 -32<br>64 -16 -24<br>32 20 -24<br>-64 -8 -28<br>-48 16 -44<br>-52 -24 -20<br>20 36 44<br>-56 -60 24<br>-64 -60 4
3	Wang, 2015	28	Evaluation of pictures. All depicted scenarios were morally good.	Moral>Gender Discrimination Control	-51 29 -2<br>45 29 -8<br>-6 50 43<br>-45 10 -41<br>24 -82 -32<br>0 47 -20<br>-3 -13 37<br>34 34 34<br>-33 -82 31<br>9 -73 58
4	Avram, 2013	16	Evaluation of morality shown in one line German poems.	Conjunction: (moral judgement > semantic control) + (aesthetic judgement > semantic	0 26 41<br>-7 27 31<br>3 14 49<br>-13 -22 -13<br>-39 26 -5<br>-51 23 7<br>-42 5 49<br>-60 -28 1

				control)	39<br>36	26<br>26	-8<br>1
				Semantic control = determining if words were real or random letter combinations	24	-91	-2
5	Han, 2016	16	Dilemma Decision	Task>Semantic Control	-8<br>-2<br>6<br>-12<br>-54<br>-2<br>56<br>16<br>-68<br>-28<br>68<br>2<br>-4<br>-36<br>-22<br>66<br>-62<br>-4<br>24<br>-26<br>-42<br>0<br>46<br>-48<br>4	40<br>-58<br>-24<br>-32<br>-74<br>4<br>-66<br>22<br>-32<br>12<br>-42<br>-50<br>-68<br>-22<br>36<br>-38<br>-28<br>54<br>32<br>-44<br>0<br>30<br>-72<br>-72<br>62	10<br>28<br>44<br>38<br>18<br>-16<br>10<br>2<br>26<br>-10<br>10<br>50<br>32<br>50<br>36<br>22<br>30<br>10<br>38<br>-22<br>8<br>28<br>18<br>14<br>30
6	Abe, 2014	25	Modified Dilemma Decision → choosing to lie or not in various real-life scenarios	Task>Semantic Control	52<br>18<br>6<br>32<br>6<br>-34<br>-24<br>-2<br>-2<br>-22	10<br>8<br>-4<br>-56<br>-60<br>46<br>-20<br>-24<br>-76<br>-78	-28<br>70<br>48<br>-2<br>0<br>30<br>76<br>26<br>30<br>-36
7	Heekeren, 2004	12	Evaluation of depicted scenario	Task>Semantic Control (does the text shown make sense or not)	55<br>-54<br>-6<br>4<br>6<br>3<br>-56<br>58	7<br>5<br>54<br>55<br>-50<br>58<br>-62<br>-60	-24<br>-26<br>-14<br>-8<br>26<br>12<br>18<br>22
8	Heekeren, 2003	8	Evaluation of	Task>Semantic	7	63	31

			text. Non-dilemma - simple/straightforward moral scenarios	Control (does the text shown make sense or not)	2<br>-8<br>-43<br>-50<br>-50<br>47<br>-57<br>7	60<br>47<br>38<br>-61<br>24<br>14<br>-14<br>-81	5<br>25<br>-10<br>19<br>-26<br>-25<br>-14<br>11
9	Prehn, 2008	23	Evaluation	Task>Semantic Control	-8<br>-40<br>-42<br>-64<br>50<br>-50<br>32<br>-14<br>20<br>46<br>42	42<br>28<br>12<br>-12<br>10<br>-68<br>70<br>-74<br>-68<br>-38<br>-82	-18<br>-22<br>-38<br>-28<br>-34<br>34<br>0<br>50<br>52<br>48<br>8
10	Schneider, 2013	28	Dilemma Decision. Asked if they agree or disagree with choice of protagonist - indicated by button press.	Task > Baseline	-3<br>-48<br>48<br>0	-55<br>-64<br>-58<br>50	34<br>22<br>22<br>22
**Subgroup = Social**							
1	Stanfield, 2017	33	Face Monitoring - Approachability Judgement	Task > gender discrimination	9<br>12<br>12<br>-36<br>30	32<br>56<br>-82<br>-70<br>-82	58<br>34<br>1<br>-44<br>-44
2	Smith, 2017	23	Face monitoring - trustworthiness judgement. Taske with deciding which out of 3 depicted faces was the most trustworthy.	Task > Perceptual discrimination control. (Choosing odd one out of the 3 faces shown)	-44<br>40<br>-8<br>-38<br>46<br>-2<br>0<br>-32<br>-44<br>6<br>-24<br>4	24<br>-76<br>-76<br>-84<br>32<br>-46<br>18<br>20<br>50<br>-12<br>-64<br>-32	28<br>-8<br>-38<br>-4<br>18<br>-36<br>50<br>-2<br>-2<br>4<br>46<br>-4
3	Hall, 2010	24	Face	Task>gender	-8	56	44

			monitoring - approachability	discrimination	-30<br>50	18<br>-6	-24<br>-34
			judgement + intelligence judgement		-52<br>30<br>18<br>-22<br>-10<br>0<br>52<br>-10<br>0<br>-48<br>20<br>-14	-24<br>-88<br>2<br>-8<br>20<br>28<br>18<br>14<br>-18<br>-66<br>-2<br>-6	-12<br>-38<br>-18<br>-20<br>54<br>2<br>42<br>10<br>-22<br>-34<br>-16<br>-18
4	Contreras, 2013	14	Asked if the depicted person or group would enjoy a given activity	Task > Perceptual control	54<br>-46<br>-4<br>-8<br>-56<br>56<br>-8<br>56<br>-42<br>-4<br>-6<br>-54<br>42<br>0	-51<br>-77<br>53<br>61<br>-9<br>-1<br>-55<br>-57<br>-63<br>47<br>45<br>-11<br>19<br>-49	16<br>30<br>-10<br>36<br>-22<br>-22<br>16<br>14<br>28<br>-8<br>50<br>-16<br>-32<br>28
5	Cunningham, 2003	15	Asked if the given famous person was ‘good’ or ‘bad’	Task>memory control: asked to determine if the person was a historically past or present figure	4<br>44<br>-12<br>44	56<br>32<br>40<br>20	20<br>-12<br>20<br>-8
6	Dolcos, 2012	18	Watch short video of social interaction. Asked to determine how trustworthy the target protagonist is	Task>Control	13<br>-53<br>48<br>52<br>-44<br>-41<br>48<br>-1<br>-45<br>-50<br>53<br>53<br>44<br>49<br>35	-3<br>-50<br>-59<br>47<br>-75<br>-63<br>-47<br>15<br>27<br>23<br>9<br>37<br>0<br>16<br>-5	-20<br>19<br>16<br>12<br>7<br>16<br>28<br>56<br>-10<br>0<br>29<br>-1<br>51<br>32<br>60

					-17<br>13<br>47	15<br>19<br>-74	5<br>5<br>3
7	Bzdok, 2012	48	Presented pair of faces. Asked to determine which is more trustworthy, attractive, happy or older.	Conjunction: (Trust>age) + (Attractiveness> age)	−3<br>−3<br>−50<br>−45<br>−3<br>−2<br>−3<br>−24<br>−33<br>−29<br>−33<br>−27<br>−35<br>33	60<br>39<br>27<br>12<br>38<br>−20<br>−48<br>−23<br>−12<br>−6<br>−6<br>−81<br>−80<br>−81	15<br>−17<br>0<br>−41<br>11<br>38<br>29<br>−18<br>−23<br>−21<br>−29<br>−39<br>−35<br>−36
8	Hensel, 2015	44	Made to listen to voices.. Asked to judge “How trustworthy?,” “How attractive?,” “How happy?,” and “How old?” are the people saying these sentences	Conjunction: (Trust>age) + (attractiveness> age)	-14<br>-56<br>62<br>52<br>-50<br>-57<br>-54<br>-38<br>60<br>33<br>2<br>-2	30<br>-57<br>-56<br>-64<br>30<br>22<br>6<br>21<br>22<br>14<br>-18<br>-87	52<br>30<br>24<br>36<br>-14<br>9<br>-36<br>46<br>10<br>-15<br>39<br>-10
9	Javor, 2018	40	Face monitoring - trustworthiness<br><br>Also tasked with evaluating trustworthiness of various brands	Task>familiarity judgement	20<br>-20<br>-30	6<br>4<br>-48	-16<br>-10<br>-16
10	Toki, 2013	30	Face monitoring - approachability judgement	Task>gender discrimination	-48<br>-48	18<br>22	10<br>-4
11	Mukherjee, 2014	24	Face monitoring - approachability judgement	Task>gender discrimination	-8<br>56<br>-52<br>30<br>-28<br>18<br>-22	40<br>24<br>18<br>-88<br>-90<br>2<br>-8	52<br>0<br>-10<br>-36<br>-34<br>-18<br>-20

12	Blasi, 2009	43	Face monitoring - approachability judgement	Task>gender discrimination	-5 18 55<br>55 13 16<br>-30 30 -19<br>36 27 -16<br>-47 13 22<br>23 -2 -24
13	Chen, 2010	21	Face monitoring - likeability judgement “do you like X?)	task>gender discrimination	-6 33 39<br>0 54 30<br>-3 54 -18<br>-6 -51 33<br>-39 21 -18<br>48 30 -12<br>45 21 45<br>-54 -60 30<br>54 -57 48<br>-21 -84 -33<br>-6 -57 -42<br>48 6 -42<br>-9 9 9
14	Rijn, 2012	18	Face monitoring - trustworthiness	Task > baseline	-48 -78 -12<br>-3 -75 0<br>48 6 36<br>30 30 -3<br>51 36 -6<br>-6 6 48<br>-33 21 0<br>9 24 30<br>-12 9 3<br>18 -24 12<br>-6 -18 9<br>-15 3 15<br>36 42 27<br>42 42 15<br>18 -6 -18<br>24 9 -9

15	Mega, 2015	28	Face monitoring - determining the authenticity of facial expressions	Task > baseline	0<br>-34<br>-28<br>-52<br>-60<br>-34<br>4<br>12<br>-34<br>-14<br>52<br>2<br>-14<br>54<br>-42<br>58<br>44<br>-52<br>30<br>6<br>-26<br>-42<br>14	-82<br>18<br>44<br>-26<br>-26<br>-90<br>34<br>-16<br>-84<br>-58<br>-26<br>50<br>-106<br>-70<br>-70<br>-42<br>-78<br>-30<br>-56<br>-78<br>-8<br>-52<br>-12	8<br>8<br>-14<br>-2<br>26<br>6<br>28<br>8<br>28<br>36<br>-4<br>16<br>-4<br>-10<br>-14<br>22<br>-2<br>22<br>-12<br>16<br>6<br>30<br>8
16	Hall, 2012	25 (all male)	Face monitoring - Approachability and intelligence judgements	Task>Baseline	-14<br>34<br>2<br>20<br>-40<br>6<br>50<br>-52<br>2	-104<br>24<br>-18<br>-102<br>24<br>30<br>10<br>-24<br>-54	-6<br>-4<br>-14<br>-6<br>-16<br>68<br>56<br>62<br>-32
17	Hall, 2012	22 (all female)	Face monitoring - Approachability and intelligence judgements	Task>baseline	38<br>26<br>44<br>-42<br>54<br>22<br>-52<br>48<br>-38	-54<br>48<br>-60<br>-64<br>36<br>32<br>-26<br>-56<br>54	-22<br>-22<br>50<br>-22<br>32<br>62<br>62<br>50<br>28
**Subgroup = Judgement under Risk**							

1	Dinu-Biringer, 2016	42	Decision making in uncertainty for reward:<br>have to select 1-5 out of 6 cards. Have to get smiley card in therefore the more cards chosen the higher the odds but lower the reward	Task > control matched for visual and motor cues	24<br>21<br>-33<br>-9<br>-24<br>15<br>-18<br>-15<br>-30<br>36<br>21<br>18<br>15<br>33<br>12<br>48	-70<br>-82<br>-10<br>14<br>-52<br>20<br>-61<br>-70<br>-34<br>59<br>-82<br>-97<br>-64<br>-91<br>-58<br>-46	-17<br>-14<br>52<br>-5<br>-14<br>-8<br>52<br>58<br>52<br>-8<br>-14<br>-5<br>-17<br>7<br>52<br>52
2	Yu, 2016	25	Balloon analogue risk task	Pumps (parametric) > control (parametric)	39<br>-39<br>6<br>9	21<br>15<br>-24<br>30	6<br>0<br>9<br>30
3	Rao, 2014	16	Balloon analogue risk task	Task > passive viewing of balloon exploding	9<br>-36	9<br>-51	48<br>45

4	Barkley-Levenson, 2013	16	Simple gamble task. All odds 50/50. Magnitude of wins and losses varied across trials	Task > Baseline	26<br>-18<br>46<br>-46<br>46<br>-58<br>10<br>-8<br>18<br>-20<br>42<br>-42<br>26<br>-18<br>52<br>-40<br>48<br>-44<br>6<br>-4<br>20<br>-22<br>42<br>-42	-90<br>-98<br>36<br>36<br>6<br>6<br>30<br>26<br>14<br>6<br>-2<br>-4<br>-90<br>-98<br>40<br>40<br>8<br>4<br>24<br>22<br>10<br>8<br>0<br>4	-12<br>0<br>20<br>20<br>26<br>30<br>20<br>28<br>-2<br>4<br>8<br>8<br>-10<br>0<br>18<br>14<br>28<br>28<br>32<br>34<br>2<br>-4<br>4<br>0

5	Galvan, 2013	43	Balloon analogue risk task	Task > control matched for visual and motor cues	4	20	38
					46	-44	44
					-40	-44	46
					34	18	-4
					-40	16	-8
					40	38	26
					-40	-50	-44
					28	6	68
					30	26	10
					-14	-74	-40
					52	-46	0
6	Congdon, 2013	23	Balloon analogue risk task	Task > baseline	2	22	38
					50	-40	52
					-40	-42	48
					50	-34	44
7	Studer, 2012	41	Roulette betting task - red and blue are varied for odds.	Task > matched control (odds are equal for red and blue)	-6	34	22
					10	28	14
					-32	30	2
					34	-48	40
					-20	-56	46
					22	-6	52
					40	53	8
					-20	-96	2
					26	-80	0
					0	-56	-34
					-42	-44	-6
					-42	-4	26
					-34	-4	52
8	Labudda, 2008	12	Simple gamble task - using dice to show odds	Task > control matched for visual and motor cues	46	34	24
					-52	18	26
					42	-46	38
					2	-72	-29
9	Ernst, 2004	17	Wheel of Fortune Task	Task > control matched for visual and motor cues	-50	16	-2
					-30	28	-8
					42	54	-8
					30	52	-16
					28	50	-12
					-20	36	-14
					28	50	-18
					24	50	-14

10	Weber, 2008	23	Binary Gamble Choice	Conjunction: Task > control + task > delay discounting task.<br><br>Control = picking larger of 2 numbers - matching for visual and motor cues	-20<br>-56<br>-28<br>-28<br>-45<br>-50<br>-3<br>22<br><br>26<br>28<br>30<br>31<br>41<br>44<br>49<br>49	-90<br>-67<br>-57<br>4<br>8<br>33<br>29<br>35<br><br>3<br>-54<br>-37<br>19<br>51<br>35<br>8<br>-59	-7<br>-8<br>47<br>52<br>30<br>17<br>35<br>-23<br><br>51<br>50<br>-7<br>-10<br>-10<br>17<br>25<br>-6
11	Vorobyev, 2015	17	Driving task in obscured vision. Decide to stop or go at various intersections	Task > baseline	-38<br><br>45<br>45<br>27<br>38<br>36<br>7<br>48<br>50<br>35<br>57<br>3<br>18<br>23<br>-61<br>54<br><br>2<br>37	13<br><br>20<br>26<br>60<br>16<br>13<br>43<br>-49<br>-40<br>-58<br>-33<br>-47<br>-40<br>-39<br>-45<br>-26<br><br>-68<br>-52	-6<br><br>16<br>-1<br>26<br>40<br>37<br>11<br>40<br>44<br>47<br>42<br>46<br>76<br>73<br>16<br>-7<br><br>4<br>-29

Subgroup = Interpersonal

1	Thompson, 2021	31	Iterated prisoners dilemma game	Decision task > baseline	6	26	46
					-42	44	-14
					45	44	25
					-48	38	22
					6	29	34
					-36	-46	40
					48	-37	46
					27	-64	40
					-24	-67	40
					12	-70	52
					-39	14	-5
					36	17	-8
					18	-10	1
					-21	-31	-2
					24	-28	-2
					15	-97	10
					18	56	31
					30	56	-11
					42	35	40
					-48	26	34
					-45	-46	52
					39	-43	43
					-24	-64	55
					27	-61	43
					-9	-76	49
					15	-4	4
					-15	-10	1
					-24	-31	1
					24	-28	1
					18	-94	10
2	Bisch, 2018	20	Iterated prisoner's dilemma game	Task > control matched for visual and motor cues	32	-54	46
					30	-60	46
					50	-46	42
					-34	-68	-30
					-32	-56	50
					44	36	28
					42	6	32
					32	22	-2
					32	58	16
					38	50	-8
					44	24	32
					6	24	50
					6	32	42
					-46	2	34
					-44	30	28
					-28	0	50
					-32	52	0
					-36	54	10
					-26	48	-12
					-6	-28	39

3	Lambert, 2017	38	Single-run prisoner's dilemma game	Task > Baseline (of first 20 trials to avoid learning effects)	-36 -2 54<br>34 2 64<br>-42 2 30<br>-8 -66 58<br>-36 -64 -8<br>10 -82 -12<br>6 18 44<br>-38 16 2<br>34 24 2
4	Bereczkei	38	2-round trust game	Task >Baseline	54 -56 -18<br>-28 -96 -6<br>30 -98 -6<br>48 10 24<br>-44 6 30<br>50 36 24<br>2 22 46<br>34 -58 48<br>-30 -58 46<br>-34 -94 -10<br>28 -100 -4<br>54 -56 -18<br>-32 24 -8<br>34 20 -6<br>38 60 2<br>46 42 30<br>-52 12 24<br>10 -8 0<br>6 30 46<br>6 -4 30<br>32 -62 46<br>-32 -56 46
5	Tomasino, 2013	13	Single-shot ultimatum game	Task > baseline	30 -46 -34<br>-14 -64 -28<br>-42 12 2<br>44 4 2<br>-12 22 -16<br>36 0 -14<br>56 -30 -14<br>-50 10 -10<br>-50 -20 50<br>-40 -26 16<br>52 -26 -16<br>-52 -66 0<br>-54 -72 14<br>-2 16 34<br>2 4 48<br>-22 16 56<br>-12 -8 72<br>6 2 66<br>24 50 26<br>14 56 24<br>-28 48 26

					26 26 38<br>24 16 40
6	Gromann, 2014	33	Iterated trust game (20 rounds)	Task > control matched for visual and motor cues	13 54 12<br>7 50 -4<br>4 -45 21<br>12 -77 -7<br>22 11 8<br>17 20 3<br>16 73 11<br>-5 59 19<br>-5 10 69<br>-12 20 2<br>-9 11 31<br>-46 -12 47
7	Corradi-Dell'Acqua, 2013	23	Ultimatum Game - Responder	Task > free win condition (matching for visual/motor cues)	42 -34 38<br>-40 -36 38<br>10 -62 10<br>-10 -66 10<br>10 -64 38<br>-8 -62 36<br>30 22 2<br>-34 16 0<br>-6 -12 -10<br>-2 18 44<br>-40 -6 52<br>36 10 54<br>42 42 14<br>50 -62 -12<br>-30 -78 -8<br>36 -84 -12<br>48 4 26
8	Emonds, 2012	23	Prisoner's Dilemma game	Task > control matched for visual/motor cues	53 11 16<br>-29 19 35<br>26 36 35<br>46 22 20<br>-31 44 22<br>27 49 23<br>-22 -3 67<br>-31 5 52<br>28 0 59<br>-8 24 39<br>-31 -53 45<br>-40 -53 45<br>37 -50 42<br>-8 -62 39<br>-10 -72 46<br>-22 -65 42<br>25 -72 31<br>-28 -71 31<br>-5 -70 61<br>-28 -75 49

					50 -55 20<br>5 -75 28<br>14 -8 52<br>5 -50 42<br>3 22 32<br>-11 -32 -1
