# Supplementary material for: Parcellation-Based Connectivity Model of the Judgement Core
Source: J Pers Med. 2023 Sep 16;13(9):1384. doi: 10.3390/jpm13091384 (PMC10532823; doi:10.3390/jpm13091384)
Supplement: Supplementary file 1 [file jpm-13-01384-s001.zip › Supplement File S3.pdf]

Supplement S3: Parcellation Based Connectivity Model of the Judgement Core

Supplement S3. Table of Correspondences (ALEs, HCP Parcellations, Networks)

	MNI	Parcellation	Network	Overlap
**Moral**	-4 -56 28	L_v23ab	DMN Core	18.41%
	-50 -62 22	L_TPOJ2	DMN PHG	12.00%
	52 10 -28	R_TE1a	DMN Temporal	7.19%
	-2 50 22	L_d32	DMN Core	7.01%
	-4 -56 28	L_d23ab	DMN Core	6.21%
	-4 -56 28	L_7m	DMN Temporal	5.92%
	-50 -62 22	L_PGi	DMN Core	5.74%
	-2 52 12	L_9m	DMN Temporal	5.58%
	-2 50 22	L_9m	DMN Temporal	4.39%
	-2 52 12	R_9m	DMN Temporal	3.12%
	-2 50 22	R_9m	DMN Temporal	3.12%
	-4 -56 28	L_31pd	DMN Core	2.90%
	-2 50 22	R_d32	DMN Core	2.48%
	-4 -56 28	R_7m	DMN Temporal	1.67%
	-4 -56 28	L_31pv	DMN Core	1.49%
	52 10 -28	R_STSva	DMN STG	1.45%
	52 10 -28	R_STSda	DMN Temporal	1.40%
	52 10 -28	R_STGa	DMN STG	0.91%

	-50 -62 22	L_STV	DMN STG	0.50%

	52 10 -28	R_TGd	DMN Temporal	0.04%
**Social**	-48 28 -12	L_47l	DMN Temporal	22.31%
	-40 22 -16	L_47s	DMN Temporal	16.53%
	-48 22 -2	L_45	DMN Temporal	12.61%
	-40 22 -16	L_47m	DMN Temporal	8.23%
	-40 22 -16	L_47l	DMN Temporal	6.30%
	18 2 -18	R_Amygdala	Limbic	5.34%
	-4 18 54	L_SCEF	Salience	4.53%
	-48 28 -12	L_47m	DMN Temporal	4.18%
	-4 18 54	L_8BM	CEN C/I	3.71%
	-4 18 54	L_SFL	DMN STG	3.02%
	-48 22 -2	L_47l	DMN Temporal	1.08%
	18 2 -18	R_EC	Limbic	0.83%
	18 2 -18	R_pOFC	Limbic	0.54%
	-48 22 -2	L_FOP4	Salience	0.49%
	-48 28 -12	L_a47r	CEN Core	0.48%
	18 2 -18	R_Pir	Sensorimotor	0.34%
	-40 22 -16	L_AVI	CEN C/I	0.13%
	-4 18 54	R_8BM	CEN C/I	0.04%
**Risk**	34 -48 40	R_IP2	CEN Lateral	24.03%

	4 22 38	R_a32pr	CEN C/I	23.46%

	34 -48 40	R_AIP	DAN	19.68%
	34 -48 40	R_LIPd	DAN	12.53%
	4 22 38	R_a24pr	Salience	11.41%
	44 36 20	R_p9-46v	CEN Lateral	8.06%
	4 22 38	R_p32pr	Salience	7.61%
	-40 14 -6	L_MI	Salience	7.57%
	48 -42 46	R_PFm	CEN Core	7.51%
	-40 14 -6	L_AAIC	CEN C/I	6.77%
	48 -42 46	R_IP2	CEN Lateral	4.21%
	44 36 20	R_IFSp	DMN Temporal	2.74%
	4 22 38	R_8BM	CEN C/I	2.54%
	44 36 20	R_46	Salience	0.03%
**Interpersonal**	2 22 32	R_a24pr	Salience	57.25%
	6 28 34	R_a32pr	CEN C/I	34.58%
	32 22 0	R_AVI	CEN C/I	27.42%
	6 28 34	R_a24pr	Salience	19.30%
	30 -62 46	R_LIPd	DAN	17.23%
	4 22 46	R_p32pr	Salience	16.92%
	4 22 46	R_8BM	CEN C/I	15.69%
	30 -62 46	R_MIP	Visual	12.03%

	-38 16 0	L_MI	Salience	11.44%

	4 22 46	R_a32pr	CEN C/I	9.79%
